# Multi-level characterization of balanced inhibitory-excitatory cortical neuron network derived from human pluripotent stem cells

**DOI:** 10.1371/journal.pone.0178533

**Published:** 2017-06-06

**Authors:** Aishwarya G. Nadadhur, Javier Emperador Melero, Marieke Meijer, Desiree Schut, Gerbren Jacobs, Ka Wan Li, J. J. Johannes Hjorth, Rhiannon M. Meredith, Ruud F. Toonen, Ronald E. Van Kesteren, August B. Smit, Matthijs Verhage, Vivi M. Heine

**Affiliations:** 1 Department of Functional Genomics, Center for Neurogenomics and Cognitive Research, Amsterdam Neuroscience, Vrije Universiteit Amsterdam, The Netherlands; 2 Department of Pediatrics / Child Neurology, Amsterdam Neuroscience, VU University Medical Center, Amsterdam, The Netherlands; 3 Department of Molecular and Cellular Neurobiology, Center for Neurogenomics and Cognitive Research, Amsterdam Neuroscience, Vrije Universiteit Amsterdam, The Netherlands; 4 Department of Integrative Neurophysiology, Center for Neurogenomics and Cognitive Research, Amsterdam Neuroscience, Vrije Universiteit Amsterdam, The Netherlands; 5 Department of Clinical Genetics, VU University Medical Center, Amsterdam, The Netherlands; 6 Department of Complex Trait Genetics, Center for Neurogenomics and Cognitive Research, Amsterdam Neuroscience, Vrije Universiteit Amsterdam, The Netherlands; Universitatsklinikum Wurzburg, GERMANY

## Abstract

Generation of neuronal cultures from induced pluripotent stem cells (hiPSCs) serve the studies of human brain disorders. However we lack neuronal networks with balanced excitatory-inhibitory activities, which are suitable for single cell analysis. We generated low-density networks of hPSC-derived GABAergic and glutamatergic cortical neurons. We used two different co-culture models with astrocytes. We show that these cultures have balanced excitatory-inhibitory synaptic identities using confocal microscopy, electrophysiological recordings, calcium imaging and mRNA analysis. These simple and robust protocols offer the opportunity for single-cell to multi-level analysis of patient hiPSC-derived cortical excitatory-inhibitory networks; thereby creating advanced tools to study disease mechanisms underlying neurodevelopmental disorders.

## Introduction

Cortical neural activity is determined by the complex interplay between excitation and inhibition [1, 2]. Distinct populations of specialized neurons originate from different neocortical regions. Excitatory projection neurons originate from cortical progenitors in the pallium [3], whereas the inhibitory interneurons originate in the ganglionic eminence (GE) of the ventral telencephalon [4]. Processes like maturation, neural specification and synapse formation all contribute to normal development of cortical networks [1]. Disruption of the balance between excitatory and inhibitory neuronal activity, leading to disturbances in network synchrony, is thought to underlie neurodevelopmental disorders, such as epilepsy, autism spectrum disorders (ASDs) and schizophrenia [5].

Patient-derived induced pluripotent stem cells (hiPSCs) hold the potential to model disease mechanisms *in vitro* [6–9], to screen therapeutic targets and to generate autologous cell populations for cell replacement therapies [10, 11]. Many differentiation protocols have been described to produce neuronal cell cultures from human pluripotent stem cells (hPSCs) or neuroepithelial stem (hNES) cells [12–16]. Several brain-patterning factors such as sonic hedgehog (SHH [17]), retinoic acid (RA [18]), fibroblast growth factors (FGFs [19]), insulin growth factors (IGFs [20]) and Wnts [21] have been used to generate specific neural cell types. Existing procedures generate mixed neural cultures, but lack derivation of pure neuronal cultures with balanced inhibitory and excitatory synaptic activities suitable for single cell analysis [22–24].

We generated low-density hPSC-derived neuronal cultures of GABAergic-glutamatergic neurons, which are amenable to multi-level analysis from early developmental to functional stages. We performed RNA expression analysis and immunocytochemistry to analyze neuronal and synaptic development, and studied functional properties by calcium imaging and patch-clamp electrophysiology. To support the maturation of neuronal precursors into functional neurons, rat astrocytes were supplemented using either a direct contact or an indirect contact co-culture system. Neuronal cell populations in the indirect co-culture mode showed no expression of glial genes, which gives new tools to study neuronal-specific changes in functional hPSC-derived cultures. These well-characterized low-density cultures will facilitate the study of disease mechanisms underlying neurodevelopmental disorders particularly involving inhibitory and excitatory network changes.

## Materials and methods

### Cell lines

H1 hESCs (male embryo), control hiPSC lines hVS-88 (74 days old male), hVS-60 (70 year old male) and hVS-421 (19 year old male) henceforth referred to as Line A, B, C and D respectively, were cultured with a feeder independent method on Geltrex in Essential 8 medium (GIBCO). The human ESC line HO1 was obtained from WiCell. The hiPSC control lines (hvs-88 and 60) were generated via reprogramming fibroblasts from two healthy individuals (fibroblasts were derived from anonymous, non-identifiable donors and therefore exempt from IRB approval). One hNES cell line was generated [25] from each stem cell line A, B and C. Repetitive differentiation experiments performed from one hNES culture are referred to as B1, B2, B3, etc.

### hNES cell generation

To obtain hNES cells, few modifications were made to the protocol described by Shi et al., 2012 [25]. In short, high-density hiPSC cultures were passaged onto Geltrex (GIBCO)-coated 12 well plates. When hiPSC cultures reached confluence, they were neural induced with Noggin (500 ng/ ml; Peprotech) or its small molecule agonist dorsomorphine (1 μM; R&D), and SB431542 (10 μM; Stegment and Selleck chemicals). Neural rosettes were picked manually and cultured in neural maintenance medium (NMM) with FGF2 (20 ng/ ml) and EGF (20 ng/ ml; Peprotech) on PLO (20 μg/ ml)/mouse laminin (20 μg/ ml; both from Sigma) pre-coated plates.

### Neuronal differentiation

hNES cells from passage 1 to 4 were used for differentiation, induced in neural induction (NI) medium [20] with SHH (400 ng/ml; Peprotech) for 4 days, and Valproic Acid (VPA; 10 μM; Sigma) for 3 days. After induction with SHH and VPA (day 8), cells were passaged with accutase (Millipore) and split at 1:2 or 1:3 (depending on the density) on PLO/ laminin pre-coated plates. Until day 17–19, cells were kept in the same well at high-density in Neurobasal medium (Neurobasal medium (Gibco, 21103–049), 1X B-27 (50X, Gibco, 17504–044), HEPES 18 mM (Gibco, 15630–056), 0.25X Glutamax (100X, Gibco, 35050–038), 100 U/ ml Pen/strep (100X, Sigma, P0781)) with BDNF (20 ng/ ml), GDNF (10 ng/ ml), IGF1 (10 ng/ ml; all from Peprotech) and cAMP (10 μM; Sigma). At this point, they were gently dissociated using Accutase. Clumps of (proliferating) cells were discarded. The single cell suspension of day 17–19 neurons was then frozen or directly plated at 62.5k cells/ 3.8 cm^2^ (one well of 12WP) in direct contact or indirect contact mode in the presence of astrocytes for maturation. To stop division of hNES cells and to facilitate neuronal maturation, the direct contact co-cultures were treated with 2 μM cytosine β-D-arabinofuranoside (AraC) at day 25 and 29. Neurons were refreshed twice a week with half of the Neurobasal medium containing BDNF, GDNF, IGF1 and cAMP. The concentration of the growth factors in the refreshment medium was doubled (enough for the whole well).

### Glial cultures

For direct contact cultures, 18 mm Menzel glass coverslips were fitted into 12 wells after alcohol sterilization and then coated by spraying with PDL-collagen-acetic acid-mix (1:1:3); PDL (0.5 mg/ ml; Sigma), collagen (3.66 mg/ ml; Corning), acetic acid (17 mM; VWR) then air dried and UV sterilized for 20 minutes. For indirect contact cultures, 12 well plates were picked with a hot needle to make little bumps in four corners of each well, and then sterilized in UV light for 20 minutes. Primary astrocytes cultured from P0 pups of pregnant female Wistar rats (From Harlan) were passaged once and frozen. When required for co-cultures, the frozen astrocytes were plated on the coated coverslips or in indirect contact wells at a density of 25k cells per 3.8 cm^2^. Astrocytes were let to grow confluent in astrocytes medium (DMEM with Glutamax (Gibco, 31966–021), 10% FCS, NEAA (100x, Sigma, M7145), Pen/strep (100x)) for about 5–6 days. At this point (day 7 of the astrocytes), neurons were plated directly on astrocyte coverslips for direct contact mode. For indirect contact mode, neurons were plated on PLO/ laminin-coated coverslips and the next day (day 19) flipped over with neuron side (down) towards astrocytes in the indirect contact wells. For indirect contact cultures, the neuron coverslips were replaced into plates with freshly plated astrocytes after 3 weeks of co-culturing. Animals were housed and bred according to institutional, Dutch and U.S. governmental guidelines. The institutional ethical committee of the VU University approved these studies.

### Immunofluorescence

Coverslips were fixed with 4% PFA or ice-cold methanol (95% methanol, 5% HAC) after 49 (direct contact) or 56 (indirect contact) days of differentiation. The immunocytochemistry staining process in short, coverslips were blocked for 1.5 hour at RT in blocking buffer (5% NGS, 0.1% BSA, 0.3% TritonX-100 in PBS 1X) then incubated with primary antibody in blocking buffer for 1 hour at RT followed by overnight incubation at 4°C (primary antibodies are listed in Table A in S1 File). After washes (3X for 5 minutes each in PBS 1X) the coverslips were incubated with secondary antibody in blocking buffer at RT for 1 hour, and mounted with Fluoromount G (Southern Biotech) on glass slides (VWR; Super frost plus). Images were acquired using a Carl Zeiss 510 Meta confocal microscope with 40x (1.2 Numerical Aperture (NA)) and 63x (1.4 NA) oil objectives. Synaptophysin1, VGAT and VGLUT1 puncta analyses were performed with 63x oil objective images using SynD [26]. A minimum of 10 fields of views (FOVs) was obtained per coverslip. And, synapses were analysed using SynD with threshold parameters of 0.5, 0.7, 1.0 and minimum size of puncta’s 0.7, 0.8, 0.8 μm for Synaptohpysin1, VGAT and VGLUT1, respectively. Immunocytochemistry was performed at RT or primary antibody incubations at 4°C and analysis was performed using Carl Zeiss 510Meta confocal or Leica DM500 B fluorescent microscope.

### Reverse transcriptase and quantitative PCR

Two—three wells (each 3.8 cm^2^) of hNES cells, hiPSCs or neurons were collected at different time points (day 5, 8, 18 and 49) and RNA isolated using trizol extraction and iso-propanol precipitation method. Equal amounts of RNA per sample were used for cDNA synthesis (1 μg). The Q-PCR experiments were performed on Light cycler 480 (Roche) equipment. The expression of each marker during differentiation days 8, 18 and 49 is normalized to the expression of the same marker at day 5. Primers used for RT- and Q-PCR are listed in Table B in S1 File.

### Calcium imaging

Day 49 (direct contact) or 56 (indirect contact) neurons were incubated for 10 minutes at 37°C, 5% CO_2_ in Fluo-5-AM ester 1 μM in neurobasal medium without growth factors and imaged under constant perfusion at a 0.4ml/minute rate with Tyrode’s solution (concentration in mM: 119 NaCl. 2.5 KCl, 2 CaCl2, 2 MgCl2, 25 HEPES, 30 Glucose) in an AxioObserver.Z1 Zeiss epifluorescence microscope using a 40X-oil objective (Numerical aperture, 1.30), 488nm excitation laser, 488±10 BP excitation filter, 525±50 emission filter and 500FT beam splitter. Images were acquired at 2Hz rate with a Hamamatsu EM-CCD camera and Axiovision 4.8 software. For drug addition, perfusion was stopped and half the volume of Tyrode’s was replaced with Tyrode’s containing the corresponding drug twice concentrated as the final working dilution. Lastly, drugs were washed away for 20 minutes at the aforementioned perfusion rate. For electrical field stimulation, the stimulation paradigm was set using a Master-8 (A.M.P.I.). 30mA pulses were generated with a stimulus isolator (World Precision Instruments) connected to two platinum electrodes placed above the region being imaged. For analysis, regions of interest (ROIs) were placed on somas and neurites based on differences between z-stack maximum and minimum projections using Image J. Duration of the calcium events and traces were subsequently determined using “Event Detection Analysis” (EvA) with in-house Matlab software scripts as described previously [27]. Drug concentrations used are listed in Table C in S1 File.

### Electrophysiology

Whole-cell recordings were performed at RT with borosilicate glass pipettes (3.5–6.5 mOhm) filled with 125 mM K^+^-gluconic acid, 10 mM NaCl, 4.6 mM MgCl_2_, 4 mM K2-ATP, 15mM creatine phosphate, 10U/ ml phosphocreatine kinase and 1mM EGTA (pH 7.30). External solution contained the following (in mM): 10 HEPES, 10 Glucose, 140 NaCl, 2.4 KCl, 4 MgCl_2_ and 4 CaCl_2_ (pH = 7.30, 300 mOsmol). Recording were acquired with an Axopatch 200A amplifier (Molecular Devices), Digidata 1322A and Clampex 9.0 software (Molecular Devices). Local field stimulation (2 mA, 1 ms) was applied with a bipolar concentric electrode placed in the vicinity of the patched neuron. Offline analysis was performed using Clampfit v9.0 (Axon Instruments) and Mini Analysis Program v6.0 (Synaptosoft).

### Mass spectrometry

Triplicate protein samples of day 56 differentiated neurons in indirect contact co-culture mode (n = 2) were collected from Line B. The cells were solubilized in 22 μL of Laemmli sample buffer. After heating at 95°C for 5 minutes, samples were loaded on a 10% SDS gel (mini-Protean 3 from Bio-Rad) and run for 45 minutes at 100 V. The gel was fixed overnight in 50% ethanol/3% phosphoric acid, stained in colloidal coomassie blue for 30 minutes, washed three times for 10 minutes in water, and each sample lane cut into two fractions and chopped into about 1 mm by 1 mm pieces. After destaining in 50 mM NH_3_ HCO_3_−50% acetonitrile/50 mM NH_3_HCO_3_ the gel pieces were dried in Speedvac, the tryptic peptides were analyzed using Triple TOF 5600+ MS (Sciex) coupled with an Ultimate 3000 LC system (Dionex, Thermo Scientific). Peptides were trapped on a 5 mm Pepmap 100 C18 column (300 μm i.d., 5μm particle size, Dionex) and fractionated on a 200 mm Alltima C18 column (300 μm i.d., 3 μm). Acetonitrile concentration in the mobile phase in 0.1% formic acid was increased from 5 to 18% in 88 minute, to 25% at 98 minute, 40% at 108 minute and to 90% in 2 minutes, at a flow rate of 400nL/minute. Peptides were electrospray into the mass spectrometer using an ion spray voltage of 5.5 kV and an interface heater temperature of 50°C. The MS survey scan range was m/z 350–1,250 acquired for 200 ms. The top 20 precursor ions were selected for 85ms per MS/MS acquisition, with a threshold of 100 counts. Dynamic exclusion was 16 s. Rolling CID function was activated, with an energy spread of 15 eV. The Human database used was UniProt_2015–02. Label-free quantification was activated with LFQ min. ratio count of 1. Match between runs with match time window of 0.7 minute and alignment time window of 4 minute were used for all analyses. For other parameters the default settings were used. All raw MS data were analyzed by MaxQuant software (version 1.5.2.8) with search engine Andromeda.

### Statistical analysis

All the statistical analysis was performed using SPSS and Prism 6 software. All raw data from Q-PCR experiments and SynD analysis were normally distributed. Statistical analysis for all the raw data was then performed using one-way ANOVA. In cases where significant differences were obtained in the ANOVA (p< 0.05) a post hoc test was performed. Post hoc tests used were Holm-Sidak's multiple comparisons test and Tukey's multiple comparisons test.

## Results

### Partial induction with SHH and VPA produces cultures with mature neuronal morphologies

**Fig 1:**
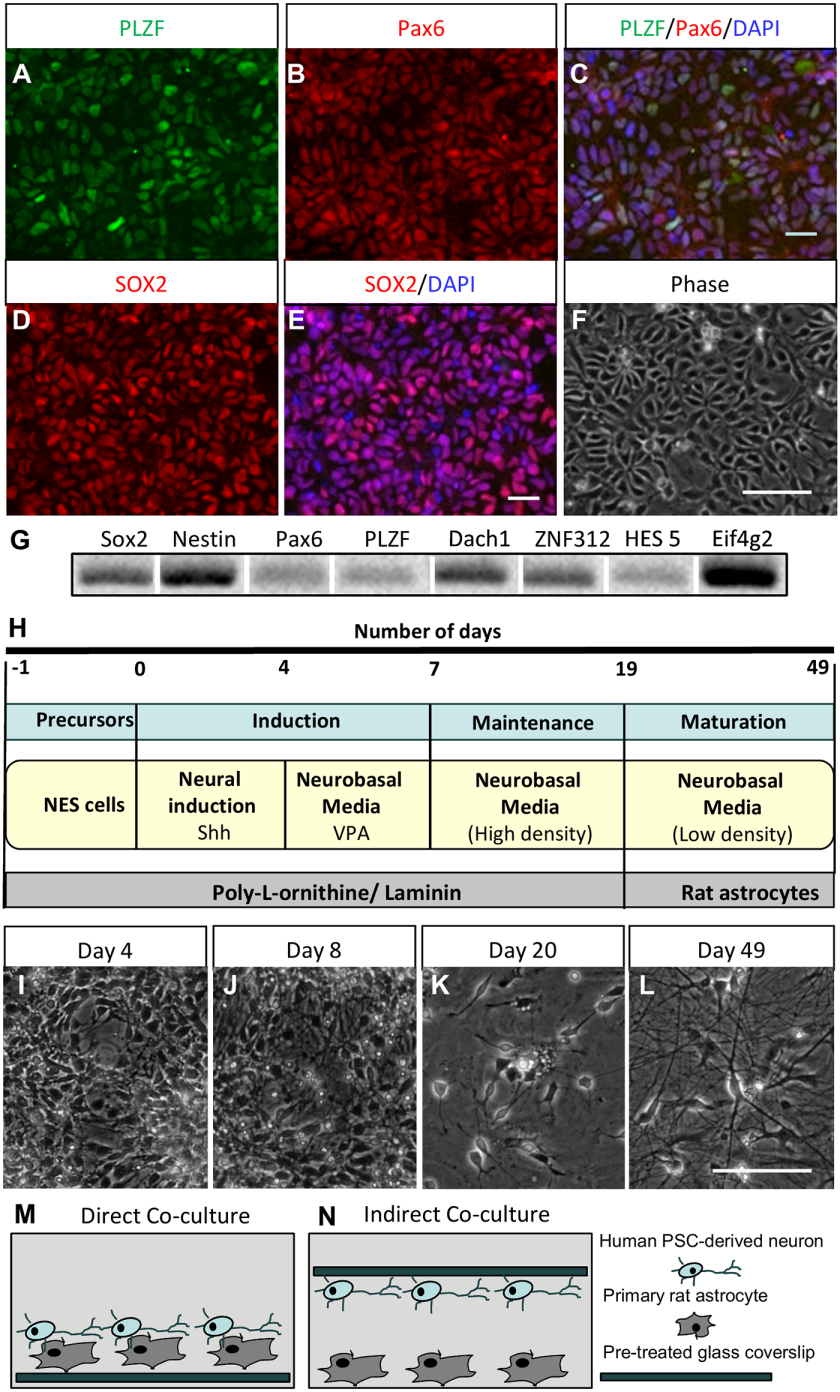
hNES cell-derived neuronal culture protocol. hiPSC-derived NES cells were generated and characterized by (A-E) immunocytochemistry for PLZF, PAX6, SOX2, (F) morphology as well as (G) RT-PCR for *SOX2*, *Nestin*, *PAX6*, *PLZF*, *DACH1*, *ZNF312* and *HES5*, and later differentiated into low-density neuronal cultures. (H) Schematic representation of the differentiation protocol. (I-L) Representative images of differentiating neurons at day 4, 8, 20 and direct contact culture mode day 49 are shown. (M, N) Cartoon representation of direct and indirect contact co-culture model. Scale bars are 25 μm. See also S1 and S2 Figs for hiPSC characterization and hESC-derived hNES cell characterization.

In order to derive a mixed culture of human GABAergic and glutamatergic neurons, hESCs and hiPSCs (S1 Fig) were neural induced into primitive rosette-shaped hNES cells as described previously [25]. Between passages 0 to 10 hPSC-derived hNES cell cultures were characterized by morphology (Fig 1F and S2F Fig) and expression of hNES cell markers [14, 28]. Immunocytochemical analysis showed that hNES cells homogeneously expressed the rosette marker PLZF, the neural precursor marker PAX6 and the stem cell marker SOX2 (Fig 1A–1E and S2A–S2E Fig). RT-PCR indicated the expression of rosette specific markers *PLZF*, *DACH1*, *ZNF312*, *HES5*, stem cell marker *SOX2*, and neural stem cell markers *Nestin* and *PAX6* (Fig 1G and S2G Fig). To generate a mixed glutamatergic and GABAergic network of neurons, a ventral GABAergic fate was partially induced using SHH [29, 30] and VPA [20, 31]. hNES cells were treated with 400ng/ml of SHH from day 0–4 followed by VPA from day 5–7 (Fig 1H). Abundant neurite outgrowth was observed at 8 days (Fig 1I). Between days 8–18 precursor cells were supplemented with growth factors BDNF, GDNF, IGF1 and second messenger cAMP to mature the neuronal cultures at a very high density (Fig 1J). Around days 17–19 highly dense neuronal precursors were dissociated, frozen and/or plated at low density to differentiate into neuronal networks (Fig 1K). To support maturation into functional neurons in low density, neuronal precursors were co-cultured with primary rat astrocytes. Human neurons (density of 62.5k cells/ 3.8 cm^2^ well) and rat astrocytes (density of 25k/ 3.8 cm^2^ well) were grown in either direct contact or indirect contact co-culture mode [32] as depicted in Fig 1M and 1N respectively. After 49 days of differentiation (Fig 1L) direct contact neuronal cultures were processed for functional analysis. Day 19 neuronal precursors, when cultured in low-density and in absence of astrocytes failed to mature and showed increased neuronal degradation around 35–40 days of differentiation. In conclusion, partial induction of hPSC-derived hNES cells with SHH and VPA produces cultures with mature neuronal morphology.

### RNA expression profiling and immunocytochemistry detects identities of glutamatergic and interneuron cell lineages of neocortical origin

**Fig 2:**
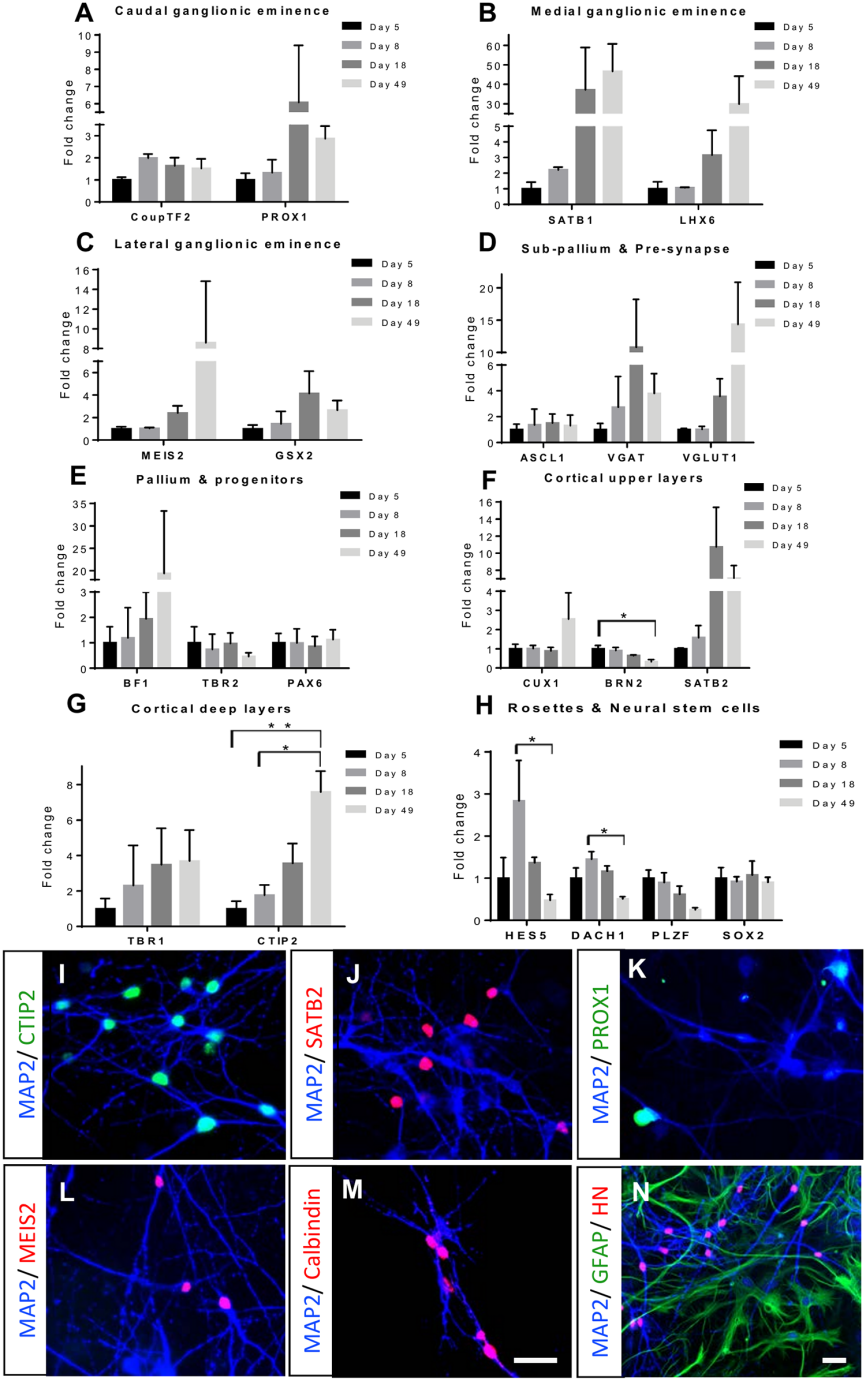
RNA and immunocytochemical analysis of hNES cell-derived neuronal cultures in direct contact. RNA was isolated at different stages of differentiation (day 5 (n = 3), day 8 (n = 2), day 18 (n = 3) and day 49 (n = 3)) altogether from Line B and C. RNA expression profiles of (A) caudal (*CoupTF2*, *PROX1*), (B) medial (*SATB1*, *LHX6*) and (C) lateral (*MEIS2*, *GSX2*) ganglionic eminence markers. (D) Expression of sub-pallium (*ASCL1*), and pre-synaptic markers (*VGAT*, *VGLUT1*) overtime during differentiation. (E) Cortical progenitors (BF1, *TBR2*) and pallium marker (*PAX6*); (F) cortical upper layers (*CUX1*, *BRN2*, *SATB2*); (G) deep cortical layers (*TBR1*, *CTIP2*) and (H) rosette and neural stem cell (*HES5*, *DACH1*, *PLZF*, *SOX2*) markers were also assessed in the RNA samples. All the primers data was normalized to the expression of housekeeping gene *EIF4G2*. Error bars represent standard mean error (SEM) per time point from n = 3 in day 5, 18, 49 and n = 2 in day 8. One-way ANOVA was performed and with significant differences (p<0.05) in some gene expressions, a post hoc test represents significant differences (in asterisks) among the time points with a p<0.05. Immunocytochemical analysis at day 49 (n = 2) for Glutamatergic lineage markers CTIP2 (I), SATB2 (J), CGE marker PROX1 (K), LGE marker MEIS2 (L) and Calbindin (M) with MAP2. (N) Only the neuronal population is human-derived as shown by immunocytochemistry for human nucleus (HN), GFAP (rat astrocytes) and MAP2. Scale bars are 25 μm.

To characterize the differentiating neurons for known markers of the pallium, sub-pallium, GE (including CGE, MGE and LGE) and cortical layers, RNA expression levels from days 5, 8, 18 and 49 (direct contact) of differentiation were analyzed. During maturation, neuronal precursors showed a gradual increase in the expression of most ganglionic eminence markers tested (Fig 2A–2C). CGE progenitor marker *CoupTF2* [33] showed a slight increase during early differentiation (day 8) and CGE marker *PROX1* [34] showed increased expression between day 5 and day 18 (Fig 2A). Although expression of MGE marker *NKX2*.*1* was not induced, other MGE markers *LHX6* and *SATB1*, respectively involved in migration of neurons from the ventral telencephalon [35] and in enhancing terminal differentiation of parvalbumin (PV) and somatostatin (SST) interneurons [36], were increasingly expressed at day 18 and 49 of differentiation (Fig 2B). Expression of LGE progenitor markers *MEIS2* [37] and *GSX2*, which regulate LGE progenitor maturation [38] showed increases in expression at day 49 and day 18, respectively (Fig 2C). The sub-pallium marker *ASCL1* stayed at a constant expression level whereas the pre-synaptic proteins *VGAT* and *VGLUT1* showed an increased expression over time (Fig 2D).

Differentiated neurons showed increased expression of several markers of cortical layer glutamatergic neurons. Throughout the differentiation, pallium marker *PAX6* showed a constant expression, cortical progenitor marker *TBR2* [39] was slightly decreased and telencephalic neuron maturation marker *BF1* [40] increased towards day 49, indicative of maturing neurons (Fig 2E). The cortical upper layer (2–4) markers *CUX1*, *SATB2* [41] showed increasing levels at day 49, whereas *BRN2* progenitor expression [42] showed a decreased level (Fig 2F). High expression of the late-onset deep layer markers *TBR1* and *CTIP2* [43] was observed at day 18 and day 49 (Fig 2G). *CTIP2* had a significant increase at day 49 compared to day 5 and day 8, indicating neural stem cells differentiation towards deep layer cortical neurons. Rosette and neural stem cell markers *HES5*, *DACH1*, *PLZF* and *SOX2* expressed at the hNES cell stage (Fig 1A–1G) showed decrease in expression during differentiation indicating neuronal maturation (Fig 2H). Specifically, neural stem cell markers *HES5* and *DACH1* showed a significant reduction from day 8 to day 49 indicating loss of neural stem cell identity. Finally, we performed immunocytochemical analysis at day 49 for markers of both lineages that showed presence of deep layer CTIP2-, upper layer SATB2-, CGE lineage PROX1-, LGE lineage MEIS2- and interneuron subpopulation marker Calbindin-positive neurons (Fig 2I–2M). To confirm that, all the neurons present in the cultures were human-derived we did a co-stain for human nuclear (HN) marker with MAP2 and astrocytic marker GFAP (Fig 2N). Taken together, RNA profiling of the developing neuronal cultures and immunocytochemistry at end stages indicate the emergence of interneurons of GE lineages, as well as glutamatergic neurons reminiscent of different neocortical layers.

### Low-density neuronal cultures generate balanced numbers of excitatory-inhibitory synapses

**Fig 3:**
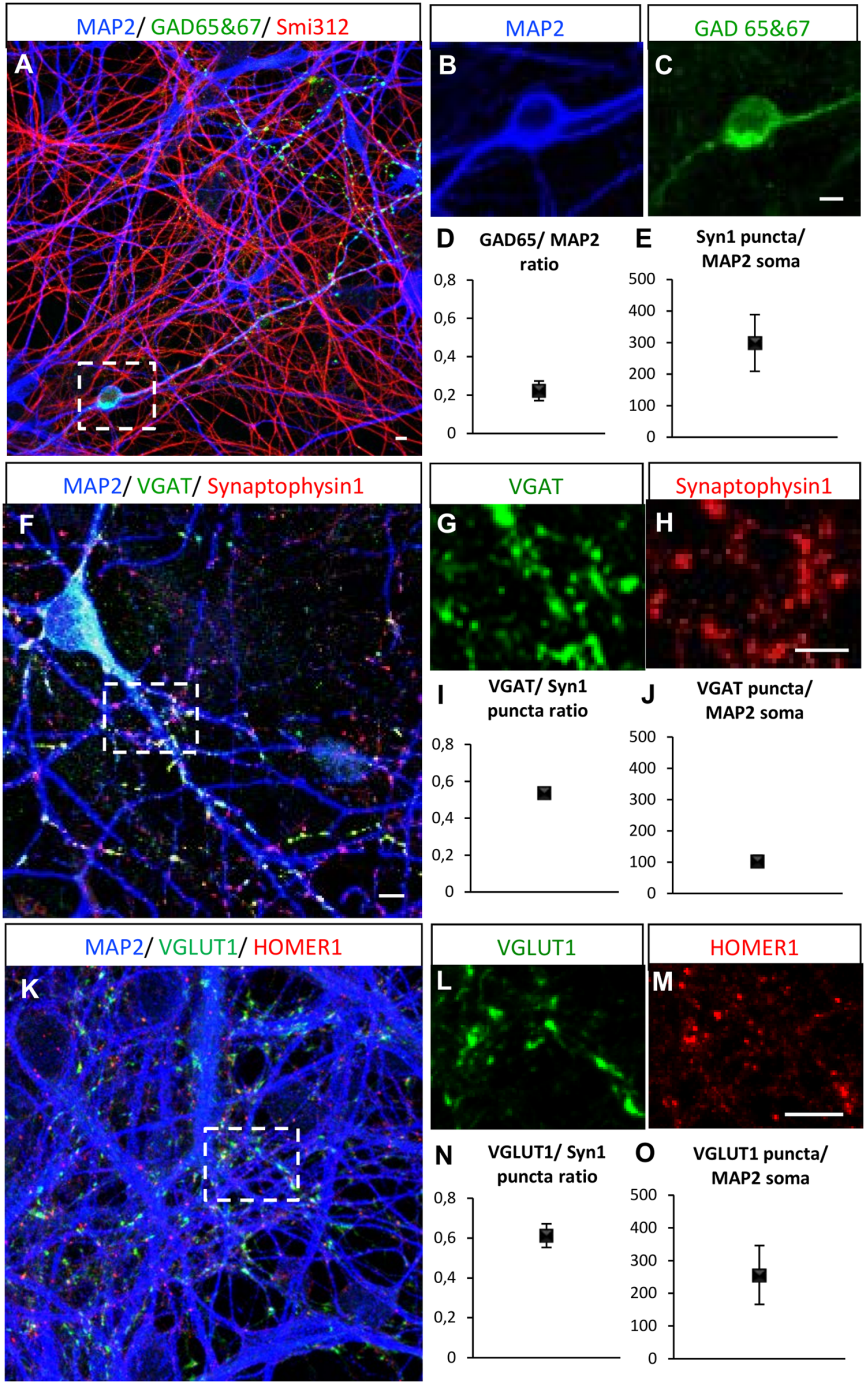
Immunocytochemical analysis of glutamatergic and GABAergic pre/post-synaptic markers in direct contact co-cultures. Immunocytochemical analysis was performed at day 49 from Line B. hPSC-derived neurons were analyzed for the expression of (A-C) MAP2, GAD65/67, SMI-312, (F-H) VGAT and synaptophysin1, (K-M) VGLUT1 and HOMER1. Ratio of (D) GAD65/MAP2-positive cells (n = 2), (E) Synaptophysin1 puncta per MAP2 soma (n = 3) (I) VGAT/synaptophysin1-positive puncta (n = 3), (J) VGAT puncta per MAP2 soma (n = 3) (N) VGLUT1/synaptophysin1-positive (n = 3) puncta and (O) VGLUT1 puncta per MAP2 soma (n = 3) (10 FOV’s per experiment) were quantified. Error bars represent SEM. Insets represent closer magnifications. One-way ANOVA was performed, no significant difference was found between experiments for GAD65 and VGAT analysis. Significant differences with p<0.05 was found between experiments B1, B2 and B3 for VGLUT1 analysis. FOV–fields of view; Scale bars are 5 μm.

To characterize excitatory and inhibitory neuron identity in the cultures, we analyzed dendritic, axonal and synaptic marker expression by immunocytochemistry. At day 49, the neuronal cultures (direct contact) showed an abundant network of dendrites and axons as shown by dendritic marker MAP2 and axonal marker Smi312 (Fig 3A). To study presence of GABAergic neurons, we quantified the number of neurons expressing the GABA-producing enzyme GAD65/67 (Fig 3A–3C) as a fraction of the MAP2-positive cells (0.22 ± 0.5; Fig 3D). We also quantified the number of synaptophysin1 puncta per neuronal soma, which accounted to 298±74 pre-synapses per MAP2 dendritic soma (Fig 3E). We further studied the presence of GABAergic presynaptic terminals by co-localization of pre-synaptic protein synaptophysin1 puncta with vesicular GABA transporter (VGAT) puncta (Fig 3F–3H). The fraction of all VGAT puncta to synaptophysin1 puncta on MAP2-positive dendrites was 0.53 ± 0.01 (Fig 3I) and the number of VGAT synapses per dendritic soma (MAP2) was 103±18 (Fig 3J). Glutamatergic neurons in the cultures were identified by co-localization of presynaptic vesicular glutamate transporter 1 (VGLUT1) with synaptophysin1 puncta, and by presence of glutamatergic post-synaptic marker HOMER1 puncta (Fig 3K–3M). The fraction of all VGLUT1 puncta to synaptophysin1 puncta on MAP2-positive dendrites was 0.61 ± 0.05 (Fig 3N) and the number of VGLUT1 synapses per dendritic soma was 255±89 (Fig 3O). These data indicate that the hPSC-derived neurons form a network with both GABAergic and glutamatergic synapses.

### Low-density neuronal cultures show synaptic activity, produce action potentials and Ca^2+^ transients

**Fig 4:**
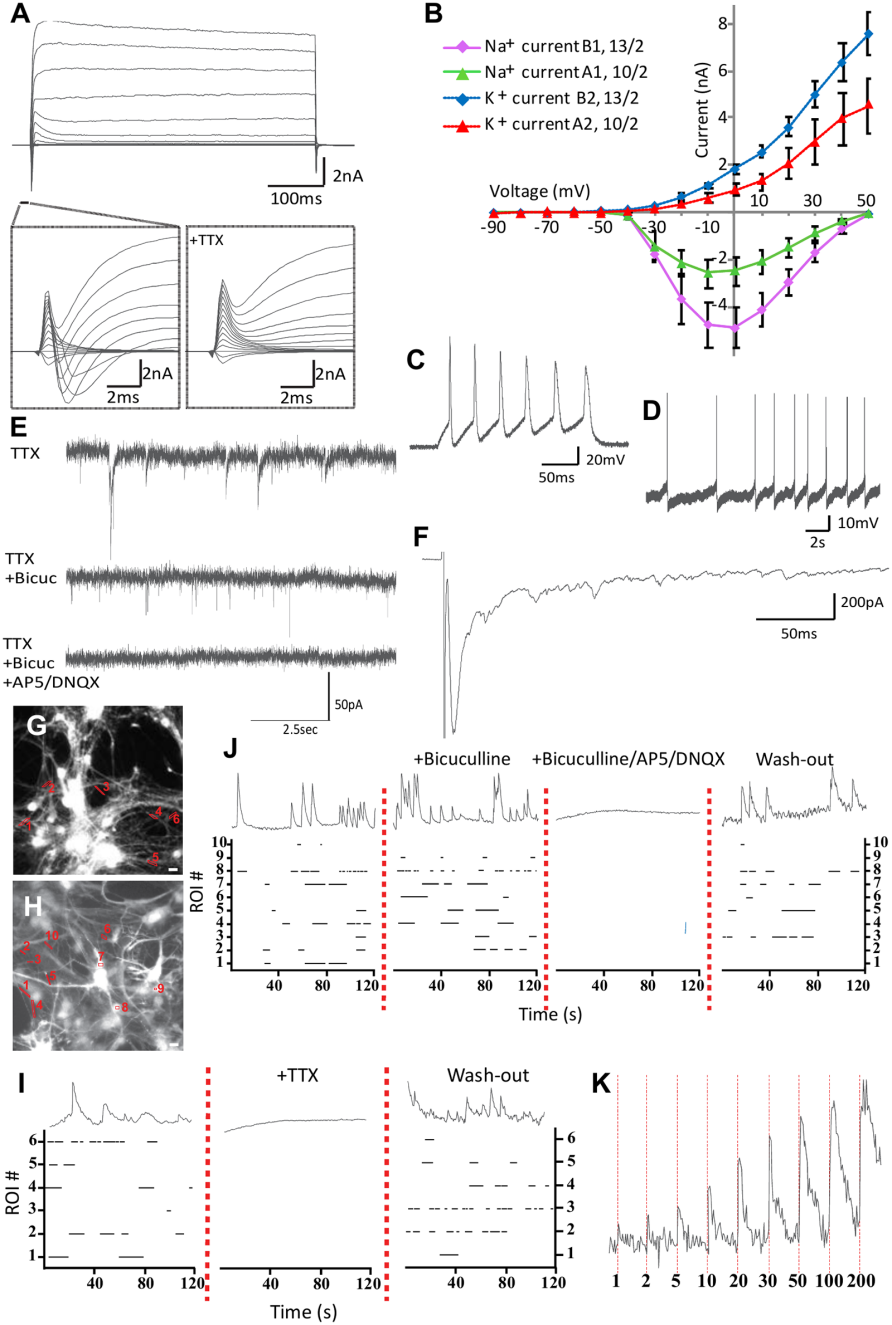
Functional analysis of differentiated neurons in direct contact co-cultures. At day 49 we recorded Line A-derived (n = 2) and Line B-derived (n = 2) neurons, which show (A) fast inward Na^+^ currents followed by long-lasting outward K^+^ currents; current was evoked by voltage steps ranging from -90 mV to 50 mV with 10 mV increments. Inset shows fast Na^+^ currents in absence or presence of Na^+^ channel antagonist TTX (1 μM). (B) Quantification of peak Na^+^ and K^+^ currents evoked in (A) is shown. (C) We recorded action potentials evoked by a current injection of 100 pA and also (D) spontaneously generated action potentials in current clamp mode. (E) Spontaneous post-synaptic events from a single neuron (holding potential at -70 mV in voltage clamp mode) recorded in presence of 1μM TTX, TTX + 40 μM Bicuculline and TTX + 40 μM Bicuculline + 50 μM AP5 + 10 μM DNQX are shown. (F) Postsynaptic current evoked by local field stimulation (2 mA, 1 ms). We recorded calcium traces at day 49 of differentiation; (G, H) hiPSC-derived neurons loaded with Fluo-5 AM ester. (I) Raster plot showing onset and duration of intracellular calcium events from ROIs represented on G; upon TTX addition and 20 minutes after TTX was washed away (J) or traces represented in H upon bicuculline, bicuculline + AP5 + CNQX addition and 20 minutes after drugs were washed away (representative calcium traces for ROI 2 (G, I) and ROI 9 (H, J)) are shown. (K) We then analyzed activity dependent intracellular calcium traces (red dotted bars indicate beginning of electrical field stimulation and number of pulses applied). Scale bars are 10 μm. Data are represented as mean ± SEM. See also S4 Fig for hESC-derived neurons calcium imaging experiments.

To understand the excitability of the differentiated neurons (direct contact), a voltage step protocol was applied to record sodium and potassium currents (Fig 4A). Quantification of the peak size of these currents showed that hiPSC-derived neurons on average had larger currents than hESC-derived neurons (Fig 4B). The presence of voltage-gated Na^+^ and K^+^ channels suggests that these neurons can fire action potentials. Indeed, the majority of cells generated action potentials in response to step current injections (Fig 4C; >90% of hiPSC-derived neurons and 70% of hESC-derived neurons). Cells unable to generate action potentials showed smaller sodium responses and responded to a smaller voltage range (-40 to -10 mV) compared to the cells that could generate action potentials (-30 to +50 mV). 30% of hESC-derived neurons and 38% of hiPSC-derived neurons spontaneously elicited action potentials in current clamp (Fig 4D). To confirm the presence of functional synapses, we assessed spontaneous activity in the presence of the voltage-gated sodium channel antagonist TTX (Fig 4E). We observed that a proportion of the spontaneous events were blocked by the GABA_A_ receptor antagonist bicuculline. Subsequent application of AMPA/NMDA receptor antagonists AP5 and DNQX blocked all remaining synaptic transmission confirming that these cultures contain both GABAergic and glutamatergic neuronal activity. To test network connectivity of the differentiated neurons, we applied local field stimulation, which elicited synchronized postsynaptic currents (Fig 4F). Hence, the majority of these hPSC-derived neurons produces action potentials and show synaptic responses.

Subsequently, we investigated the capability of the neurons to generate spontaneous activity in these networks by monitoring Ca^2+^ transients. We loaded the neurons with Fluo-5-AM and imaged Ca^2+^ transients in regions of interest (ROIs) on somas and neurites. We observed abundant spontaneous network activity, which was blocked by TTX (Fig 4G, 4I, 4C and S4A Fig) and re-established after wash out, except for a few ROIs (17% for both hiPSC- and hESC-derived neurons). Addition of bicuculline triggered a change in spontaneous activity (Fig 4H, 4J, 4D and S4B Fig) with some neurons increasing (>60% increase in 15% of hiPSC- and in 25% of hESC-derived neurons) and others decreasing frequency of events (>60% reduction in 50% of hiPSC- and in 40% of hESC-derived neurons). Subsequent addition of AP5 and DNQX blocked spontaneous calcium transients in all hiPSC-derived neurons, but remained in 10% of the hESC-derived neurons, suggestive of intrinsic pacemaker activity in a small group of neurons. The network also responded to electrical field stimulation with increased activity/amplitude correlated to the number of pulses given (Fig 4K and S4E Fig). These results indicate that the hPSC-derived neurons trigger action potentials, establish active glutamatergic and GABAergic synapses and generate synaptic transmission in the neuronal network.

### Indirect contact co-cultures of neurons form excitatory-inhibitory synapses

**Fig 5:**
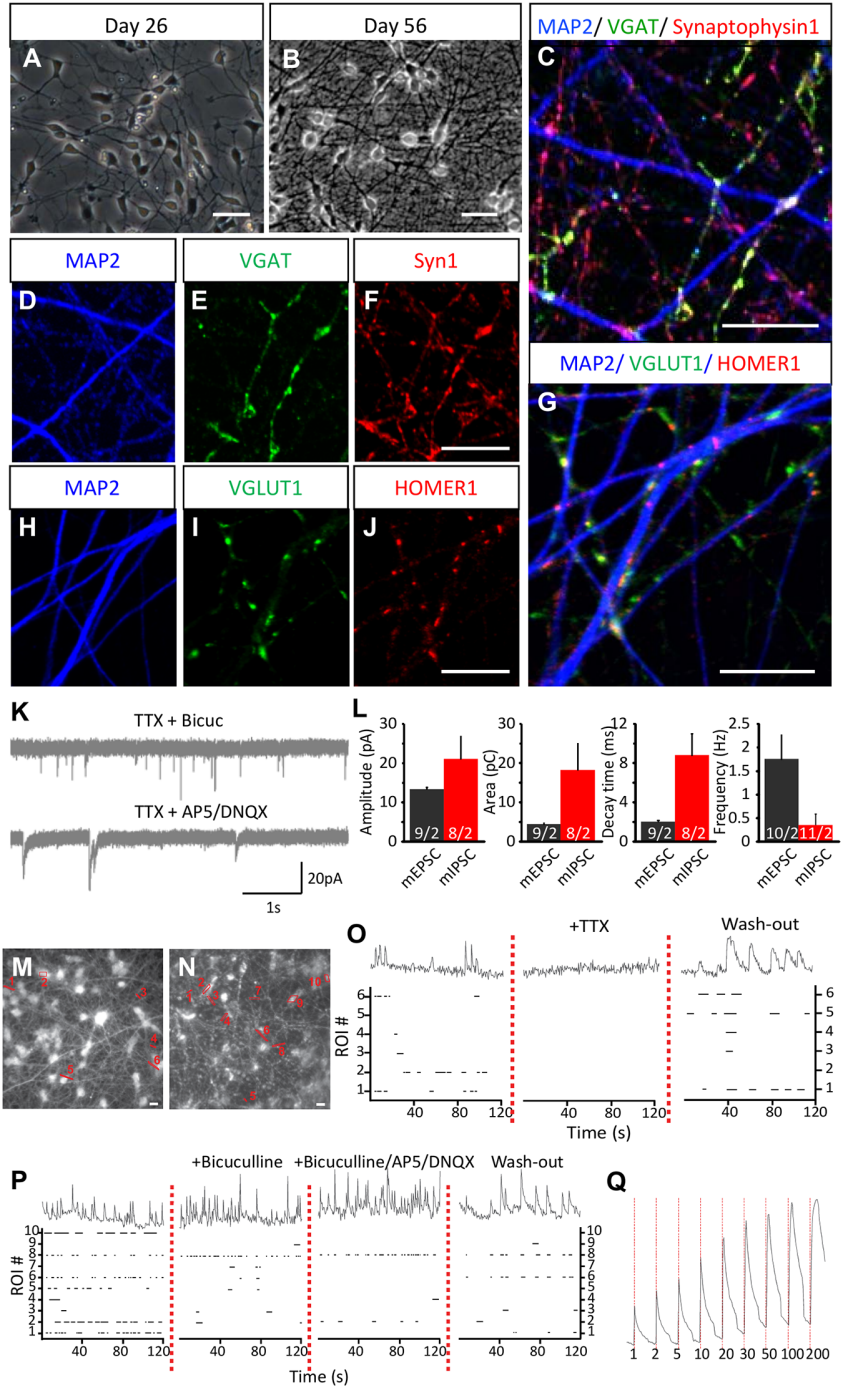
Characterization of the differentiated neurons in indirect contact co-cultures. In indirect co-cultures we differentiated the human neurons in close proximity of rat astrocyte layers (Line B1 and B2). (A, B) Bright field images of indirect cultures during differentiation day 26 and 56 are shown. (C-F) We observed the expression of MAP2, VGAT, synaptophysin1 and (G-J) VGLUT1 and HOMER1 co-localizing puncta using immunocytochemistry at day 56. We then analyzed (K) spontaneous mEPSCs and mIPSCs (holding potential at -70mV in voltage clamp mode) recorded in 1 μM TTX supplemented with 40 μM Bicuculline or 50 μM AP5 + 10 μM DNQX respectively. (L) Quantification of amplitude, area, time to decay and frequency of mEPSCs and mIPSCs was also analyzed (n = 2). We then recorded calcium traces of (M, N) neurons loaded with Fluo-5 AM ester from n = 2. (O) Raster plot showing onset and duration of intracellular calcium events from ROIs represented on M and N respectively; upon TTX addition and 20 minutes after TTX was washed away (P) or upon bicuculline, bicuculline + AP5 + CNQX addition and 20 minutes after drugs were washed away (representative calcium traces for ROI 1(M, O) and ROI 8 (N, P)) are shown. (Q) Analysis of activity dependent intracellular calcium traces (red dotted bars indicate beginning of electrical field stimulation and number of pulses applied). Scale bars are 10 μm. Data are represented as mean ± SEM. See also S5 Fig for indirect contact plate preparation method.

To facilitate neuronal specific analysis in the absence of astrocytes, we grew neurons in indirect contact with astrocytes. These cultures manifested morphology of very well connected neurons at early (day 26; Fig 5A) and later differentiated stages (day 56; Fig 5B). At day 56, VGAT and synaptophysin1 (Fig 5C–5F) as well as VGLUT1 and HOMER1 (Fig 5G–5J) were expressed in a punctate pattern, suggesting the presence of GABAergic and glutamatergic synapses. Similar to the direct contact cultures, the indirect contact cultures were positive for the lineage identity markers CTIP2, SATB2, PROX1, MEIS2 and Calbindin at day 56 using immunocytochemistry (S3I–S3M Fig). Indeed, these neurons established GABAergic and glutamatergic synapses as measured by whole-cell patch clamp electrophysiological recordings of spontaneous release (Fig 5K and 5L). Furthermore, Ca^2+^ transients in indirect contact cultures demonstrated a functionally integrated network (Fig 5M and 5O) of mixed neurons (Fig 5N and 5P), which showed increased amplitude of response to an increasing number of stimuli (Fig 5Q). We also observed ROIs, which upon bicuculline addition showed either increased (20%) or decreased (55%) frequency of activity, and upon bicuculline/AP5/DNQX addition showed persistent activity (10%) and did not recover activity after TTX wash out (17%). Finally, to study differences between direct and indirect contact cultures, we analyzed RNA of both neuronal cultures at end stages for lineage markers of GABAergic and glutamatergic neurons. There are no significant differences in the expression of markers between direct and indirect contact cultures (Fig 3A–3H). Although we need to consider that the end-stage analysis of direct and indirect cultures was at different time points (day 49 and day 56). These observations confirm that hPSC-derived neurons also form a functional and mixed synaptic network of GABAergic and glutamatergic neurons, when grown with rodent astrocytes in an indirect contact co-culture system.

### Proteomic analysis of indirect contact co-culture neurons shows low variation between replicates

**Fig 6:**
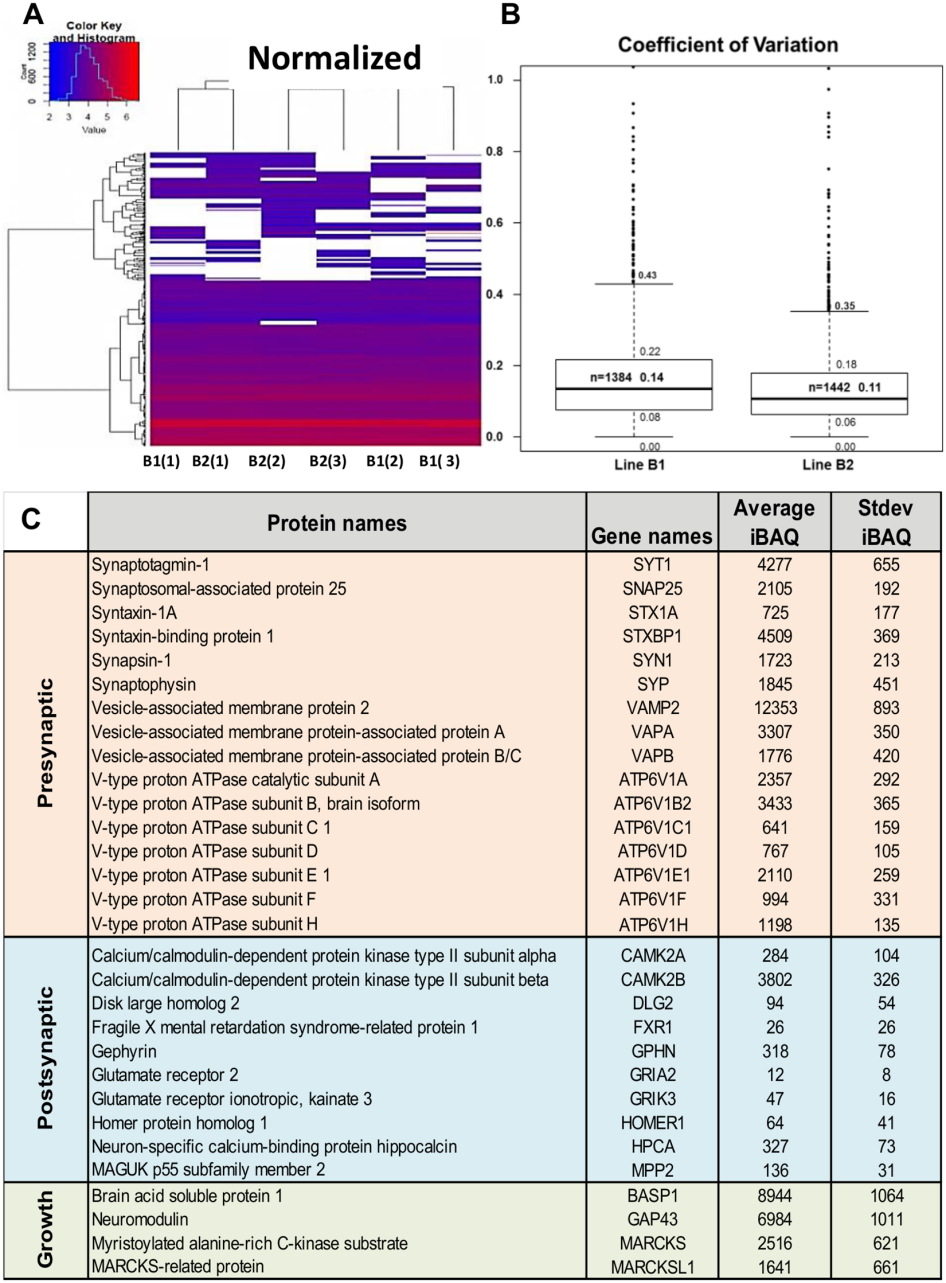
Proteomic analysis of indirect contact co-culture neurons representing reproducible cultures. (A) Hierarchical clustering of protein groups based on expression intensity present in the day 56 differentiated neurons. Columns represent triplicates from 2 experiments (B1 and B2) normalized for loading and appear similar in highly expressed proteins (color key values > 4). (B) Average of the coefficient of variation normalized to loading for all the proteins. (C) Average and standard deviations of intensity based absolute quantification (iBAQ) values for pre-, post-synaptic and growth cone proteins in day 56 neuronal cultures.

To evaluate the reproducibility of the neuronal differentiation and to check if the indirect contact co-cultures produced pure neuronal batches, we performed mass spectrometry analysis. At day 56, we collected whole cell lysates from triplicate samples of two hiPSC-derived differentiations (namely line B1 and line B2). In line B1, 1384 proteins were detected in 3 replicates; in line B2, 1442 proteins were observed in 3 replicates. Overall the protein expression profiles between samples as analyzed by hierarchical clustering (Fig 6A), were very similar indicative of corresponding expression profiles between the different cell cultures. The average coefficient of variation of each sample normalized with respect to the loading was small, ranging between 0.11–0.14 (Fig 6B) indicating similarity of protein expression between replicates. Typical neuronal-specific proteins, such as MAP2, Beta-3 tubulin, Syntaxin, Synapsin, Synaptotagmin and Gephyrin were detected (S1 Table). Proteins were analyzed for differences in expression by using the average IBAQ values of the 3 technical replicates per line. This revealed the abundant presence (high IBAQ values) of presynaptic and growth cone proteins (Fig 6C) such as Synaptophysin, SNAP25 and Neuromodulin. Glial proteins such as GFAP, ALDH1L1, GLAST, O4 and PDGFaR were not detectable (S1 Table), which indicates that by indirect contact co-cultures one can generate pure neuronal cell cultures. Taken together, these results indicate little variation in protein expression of whole cell lysates between independent experiments and confirmed absence of glial cells in the neuronal cultures.

## Discussion

We present a robust and simple hPSC differentiation protocol to derive a low-density culture of cortical glutamatergic and GABAergic neurons, following *in vivo* developmental processes. Using electrophysiology, calcium imaging, confocal microscopy, mRNA and proteome analysis, we proved functional activity of excitatory-inhibitory networks with reproducible synaptic balance. Although earlier studies produced mixed neuronal cultures, we generated a low-density network with purely cortical neurons. These cultures facilitate the analysis of inhibitory and excitatory network changes in patient hiPSC-derived cortical networks at single cell resolution.

To generate complex neuronal networks representative of *in vivo* cortical brain circuitries both inhibitory interneurons and excitatory projection neurons should be present. To induce interneuron cell fate, we treated the high-density hPSC-derived NES cells with SHH and VPA. Previous studies showed that SHH ventralizes the neural tube along the anterior-posterior neural axis [29] and thereby induces cortical interneuron specification [44], whereas VPA administration to human and rodent PSC cultures increases the expression of the GABA-synthesizing enzyme GAD65/67 [20, 31]. To induce pure cultures of hPSC-derived GABAergic neurons, Ma and colleagues used extended treatments with SHH (200ng/ml) for 14 days and VPA (10 μM) for 7 days [20]. Since we aimed for induction into ventral interneuron cell fate in only a subset of the progenitors, we tested short-treatment procedures with SHH (400 ng/ml for 4 days) and retained dorsal identities to achieve spontaneous differentiation of glutamatergic neurons as well. To confirm generation of GABAergic and glutamatergic neurons following *in vivo* development [3, 44], we performed RNA expression profile analyses at different time points of differentiation and immunocytochemistry analysis at the end stage. In our neuronal cultures, the interneuron population consists of CGE- and LGE-derived cells as indicated by expression of post-mitotic markers *PROX1* and *MEIS2*. The cultures also express MGE markers *SATB1* and *LHX6*, but lack MGE marker *NKX2*.*1*. Since our RNA profiling analysis did not detect *NKX2*.*1*, we also did not expect Parvalbumin (PV)- and Somatostatin (STT)-positive neurons, as previous studies showed that PV and STT is reduced in Nkx2.1 KO mice [45]. We found expression of other GE lineage interneuron markers, like Calbindin in both direct and indirect contact co-cultures. The excitatory neurons were representatives of upper and deep neocortical layer cells, as shown by expression of the post-mitotic markers CUX1, SATB2, TBR1 and CTIP2, respectively. We successfully generated and characterized a protocol to produce a mixture of both cortical glutamatergic and GABAergic neurons in line with *in vivo* development.

A reproducible balance in excitatory and inhibitory activity will facilitate the study of cortical networks and neuronal communication. We characterized and quantified the number of pre- and post-synaptic terminals. End-stage direct cultures demonstrated the presence of 50–55% GABAergic (VGAT) and 50–67% glutamatergic (VGLUT1) pre-synaptic terminals and 17–27% of neurons expressed GAD65/67, which is representative of human cortical development [46]. A subset of the glutamatergic post-synaptic HOMER1-positive puncta appeared VGLUT1-negative and may express other glutamatergic transporters like VGLUT2 [47, 48]. Since indirect cultures did not receive AraC treatment, we suspect that the implementation of AraC treatments increases maturity levels of these cultures. The hPSC-derived neuronal cultures manifested functional neurons by presence of evoked and spontaneous action potentials as well as miPSCs and mEPSCs mediated by GABA and NMDA/AMPA receptors, respectively in both co-cultures. The peak size of sodium currents showed that hiPSC-derived neurons had on average larger currents than hESC-derived neurons, suggestive of a higher maturation state, as reported previously [49]. When spontaneous calcium transients were measured, few ROIs increased activity upon TTX wash-out, likely due to a rebound effect of this compound [50]. Bicuculline administration changed calcium activity indicating functional neurons, as GABAergic neurons are excitatory during development. Kuijlaars *et al*.[51] recently presented protocols of hiPSC-derived cortical neuronal cells in co-culture with primary human astrocytes. Interestingly, they found increased maturation of co-culture systems around day 80 compared to neuronal monocultures by studying synchronized calcium influx activity. This confirms that astrocytes are of utmost importance to generate functional neuronal networks. Further studies should show when our co-culture systems show synchronized activity. Overall, our immunocytochemical and electrophysiological analyses indicate derivation of neuronal networks with mixed glutamatergic and GABAergic neuronal activity.

Astrocytes are important players in neuronal maturation and activity [52, 53]. Maturation of precursors into functional neurons is therefore regularly facilitated by co-culturing with astrocytes [54]. To identify neuronal-specific changes, removing astrocytes at the analysis stages is however often desired. While we expect that astrocyte-conditioned media will not be sufficient to support neuronal maturation of low-density culture of purely neurons, we have not explored these culture settings. In the direct and indirect contact cultures used for this study, we did not observe astrocyte loss. Furthermore, we were able to generate pure hPSC-derived neuronal networks when neuronal precursors were co-cultured with rat astrocytes in the indirect contact mode. This was confirmed by mass spectrometric analysis, which revealed no presence of human glial proteins after removal of the astrocytes. When we cultured neurons in complete absence of astrocytes we noticed generation of glial cells from neuronal precursor cells. This indicates that neuronal precursors that are maturing towards functional pure neuronal networks need presence of astrocytes.

Reproducibility and consistency of protocols is a major limitation in the stem cell differentiation field. We performed one of the first studies using mass spectrometry to test reproducibility of hPSC-derived neuronal differentiation procedures. Inspecting the protein profiles of the end-stage neurons showed limited intra-line variability, as illustrated by growth cone proteins, neuronal (like MAP2 and TUBB3) and pre-synaptic proteins (like synapsin and synaptotagmin) analyzed. So we confirmed reproducibility of our hPSC differentiation procedures, showed that mass spectrometric analysis of indirect contact cultures will facilitate neuronal- (or astrocyte-) specific protein analysis and gives important tools to get insight into changes in overall maturity levels of neuronal networks. Although, more in depth proteomic studies might be required to extrapolate our findings to other hiPSC lines.

In conclusion, we derived well-characterized reproducible low-density neuronal cultures from hPSCs with inhibitory and excitatory neuronal activity, which represent development of human cortical neurons. These protocols provide new tools to study cortical neuronal network changes at single cell stage as well as network level. This will facilitate studies of disease mechanisms involving imbalance between excitatory and inhibitory activity, which is thought to underlie different neurodevelopmental disorders.

## Supporting information

S1 Fig hiPSCs characterization. (TIF)

S2 Fig hESC-derived NES cells. (TIF)

S3 Fig Comparison of RNA expression profile between direct and indirect co-cultures and protein profile expression of indirect co-cultures. (TIF)

S4 Fig Calcium imaging of hESC-derived neurons in direct co-cultures. (TIF)

S5 Fig Sandwich astrocyte plate preparation. (TIF)

S1 Table Proteomic analysis. (XLSX)

S1 File Supplemental file. (DOCX)
