# Supplementary figures and images for: Multi-level characterization of balanced inhibitory-excitatory cortical neuron network derived from human pluripotent stem cells
Source: PLoS One. 2017 Jun 6;12(6):e0178533. doi: 10.1371/journal.pone.0178533 (PMC5460818; doi:10.1371/journal.pone.0178533)

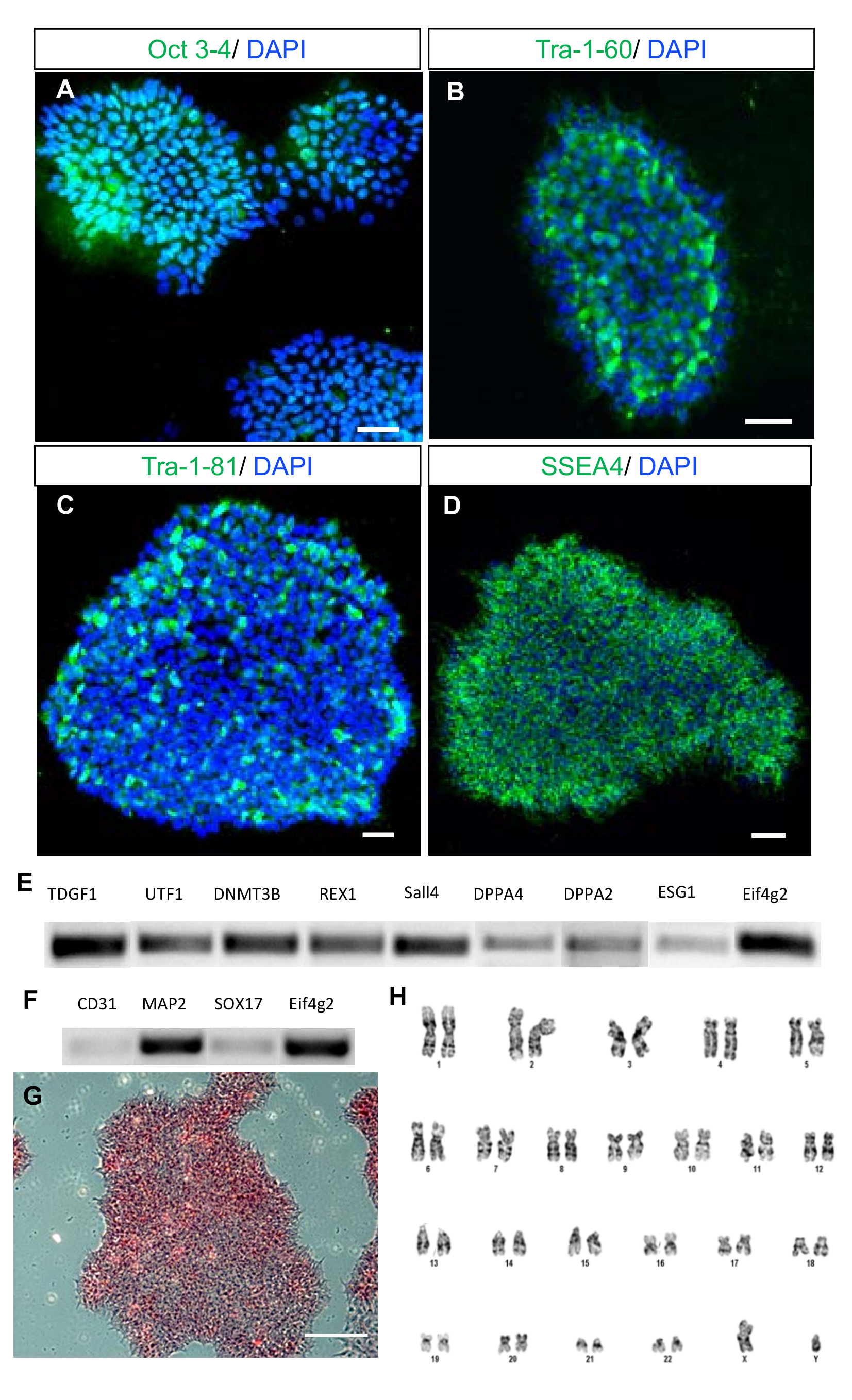

Supplement: S1 Fig — (TIF) [file pone.0178533.s001.tif]

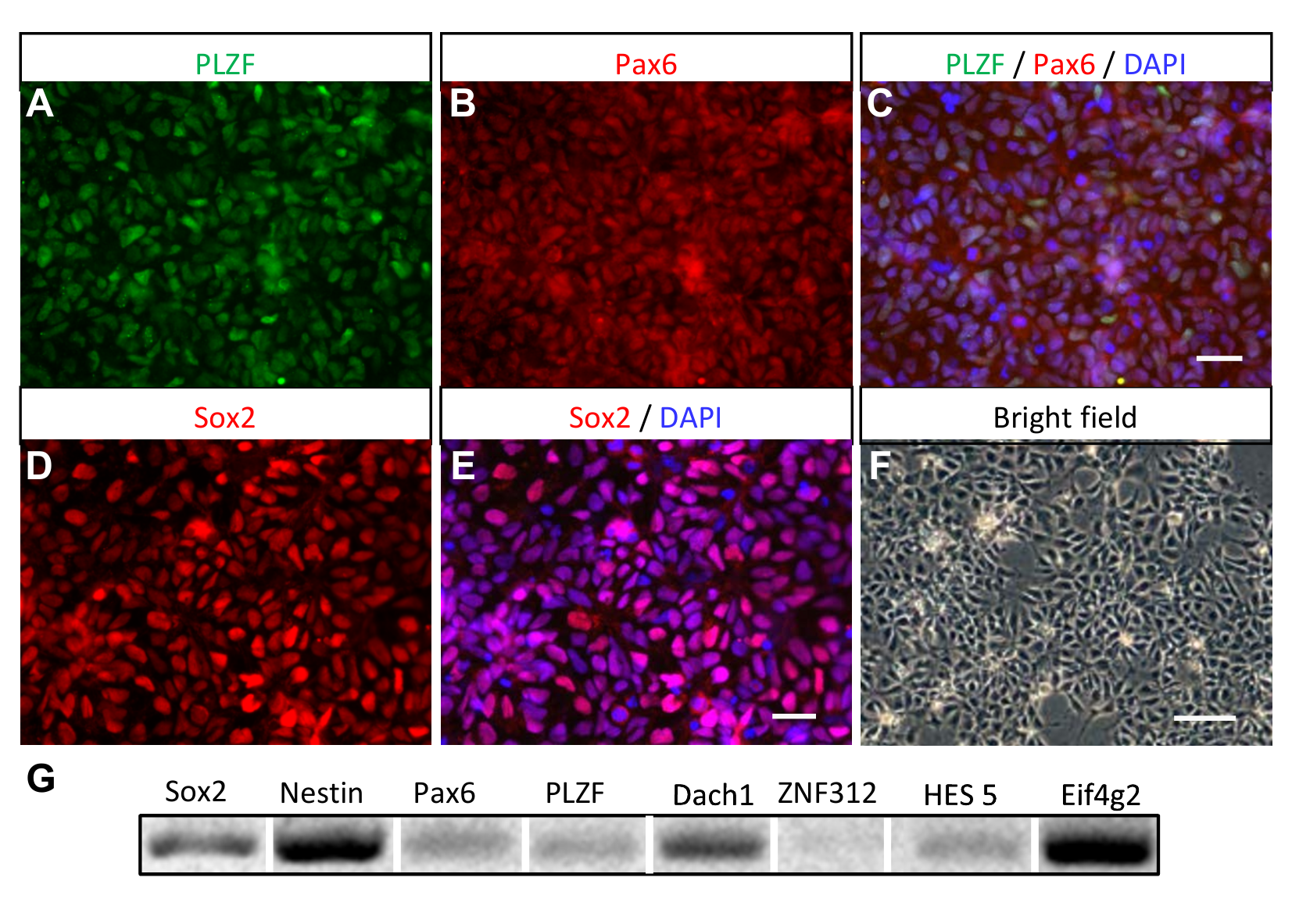

Supplement: S2 Fig — (TIF) [file pone.0178533.s002.tif]

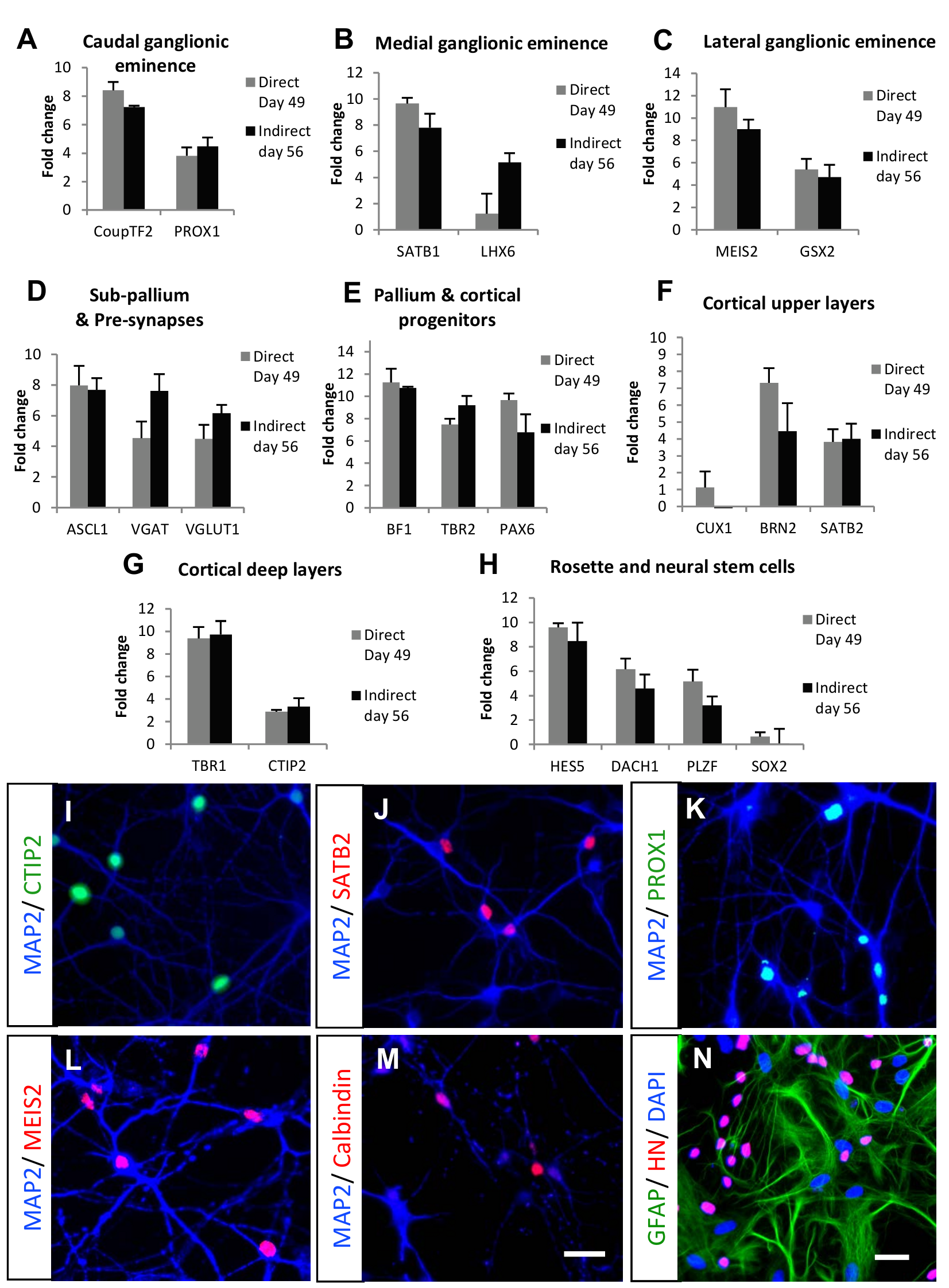

Supplement: S3 Fig — (TIF) [file pone.0178533.s003.tif]

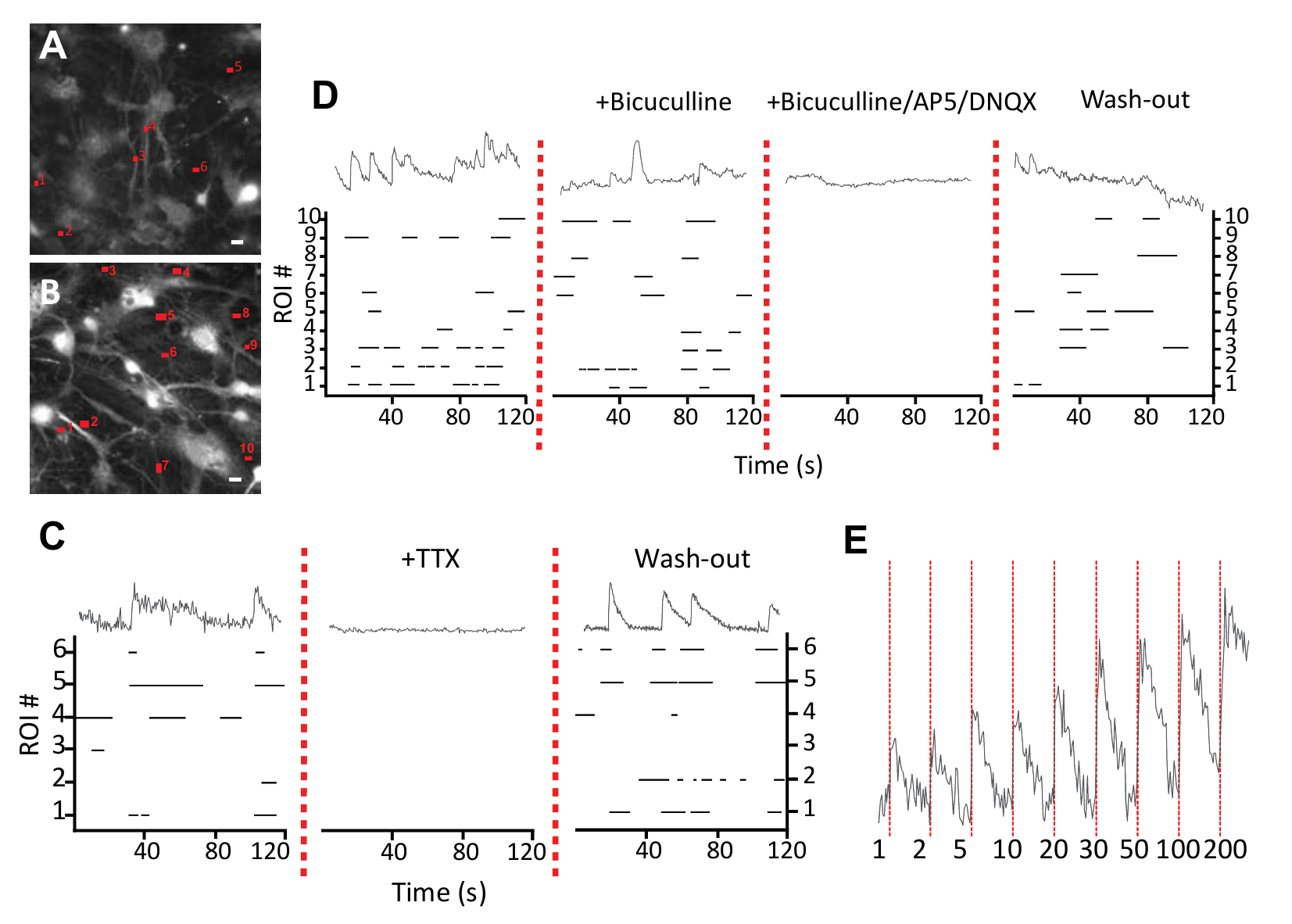

Supplement: S4 Fig — (TIF) [file pone.0178533.s004.tif]

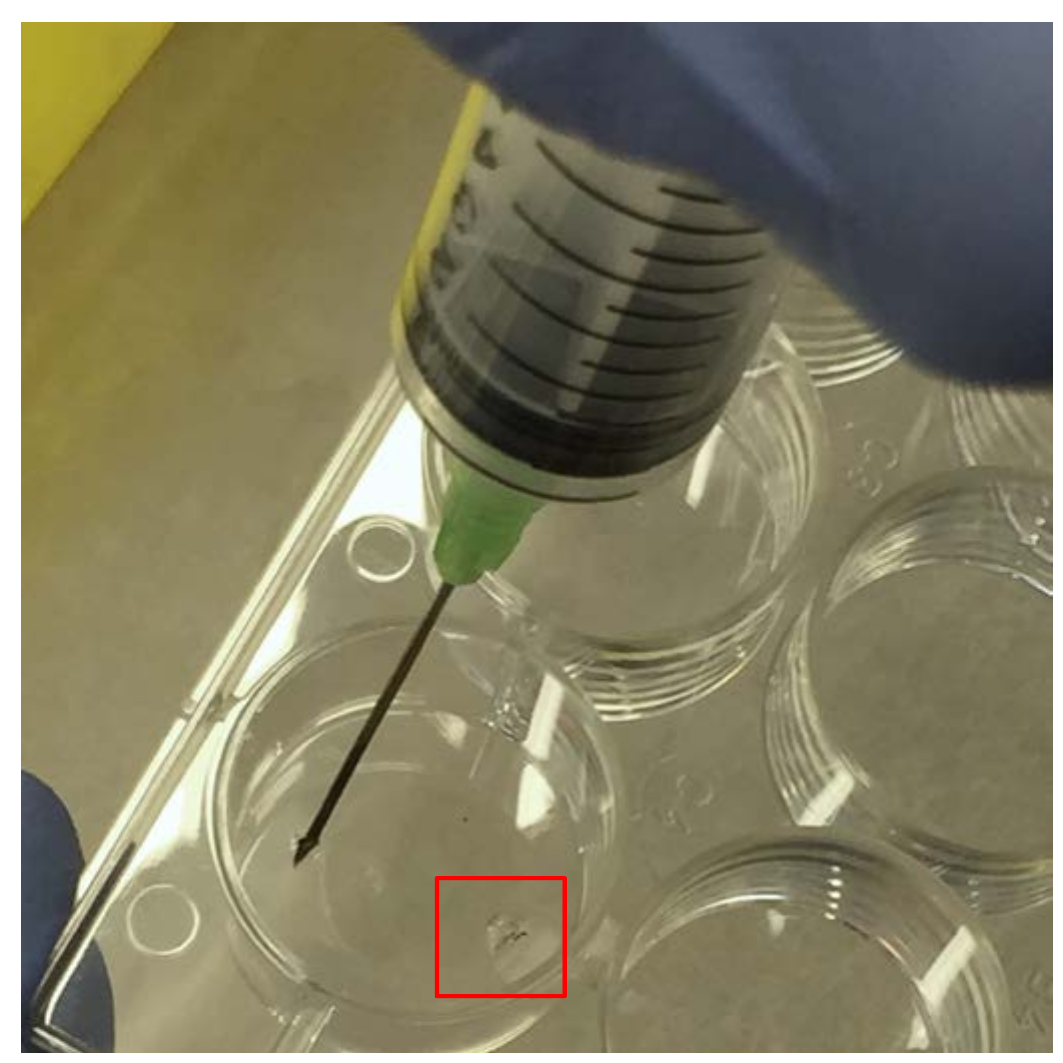

Supplement: S5 Fig — (TIF) [file pone.0178533.s005.tif]
