# Supplementary material for: Multi-level characterization of balanced inhibitory-excitatory cortical neuron network derived from human pluripotent stem cells
Source: PLoS One. 2017 Jun 6;12(6):e0178533. doi: 10.1371/journal.pone.0178533 (PMC5460818; doi:10.1371/journal.pone.0178533)
Supplement: S1 File — (DOCX) [file pone.0178533.s007.docx]

**Supplemental figures legends**

**Figure S1 related to Figure 1 – hiPSC characterization.** hiPSC clones derived from controls were fixed in PFA and immunostained for (A-D) stem cell/ pluripotency markers OCT3/4, Tra-1-60, Tra-1-81 and SSEA4. (E) RT-PCR analysis for stem cell markers *TDGF1, UTF1, DNMT3B, REX1, Sall4, DPPA4, DPPA2, ESG1* and reference gene *Eif4g2.* (F) hiPSCs were differentiated to 3 germ layers expressing *CD31* (mesoderm)*, MAP2* (ectoderm) and *SOX17* (endoderm). (G) Alkaline phosphatase stained hiPSC colonies in bright field. (H) Karyotype of the hiPSC clone. Scale bars are 50 μm.

F**igure S2 related to Figure 1** – **hESC-derived NES cells.** NES were generated from hESCs, characterized by immunocytochemistry for (A-E) PLZF, PAX6, SOX2 (passage 6), (F) morphology (passage 0) and (G) by RT-PCR for the markers *SOX2, Nestin, PAX6, PLZF, DACH1, ZNF312* and *HES5* (passage 7) and later differentiated into low density neuronal cultures. Scale bars are 25 μm.

**Figure S3 related to Figure 2 – Comparison of RNA expression profile between direct and indirect co-cultures and protein profile expression of indirect co-cultures.** RNA was isolated from direct co-cultures at day 49 (n=3) altogether from Line B and C and indirect co-cultures at day 56 (n=2 from Line B). Comparative RNA expression profiles of (A) Caudal (*CoupTF2, PROX1*), (B) medial (*SATB1, LHX6*) and (C) lateral (*MEIS2, GSX2*) ganglionic eminence markers. (D) Expression of sub-pallium (*ASCL1*), and pre-synaptic markers (*VGAT, VGLUT1*). (E) Comparative expression of cortical progenitors (BF1, *TBR2*) and pallium marker (*PAX6*); (F) cortical upper layers (*CUX1, BRN2, SATB2*); (G) deep cortical layers (*TBR1, CTIP2*) and (H) rosette and neural stem cell (*HES5, DACH1, PLZF, SOX2*) markers were also assessed in the RNA samples. All the primers data was normalized to the expression of housekeeping gene Eif4g2 and final ratio were normalized to expression of different genes at iPSCs stage. Error bars represent standard mean error (SEM) per time point from n=3 in direct day 49 and n=2 in indirect day 56. Immunocytochemical analysis at day 56 (n=2) of indirect co-cultures for Glutamatergic lineage markers CTIP2 (I), SATB2 (J), GABAergic lineage markers PROX1 (K), MEIS2 (L) and Calbindin (M) with MAP2. (N) Representation of GFAP, human nucleus (HN) and DAPI stain in direct contact co-cultures. Scale bars are 25 μm.

**Figure S4 related to Figure 4 – Calcium imaging of hESC-derived neurons in continental co-cultures.** We recorded calcium traces at 49 days of differentiation; (A, B) hESC-derived neurons loaded with Fluo-5 AM ester. (C) Raster plot showing onset and duration of intracellular calcium events from ROIs represented on A&B respectively; upon TTX addition and 20 minutes after TTX was washed away (D) or upon bicuculline, bicuculline + AP5 + CNQX addition and 20 minutes after drugs were washed away (representative calcium traces for ROI 1 (A, C) and ROI 1 (B, D)) are shown. (E) Activity dependent intracellular calcium traces (red dotted bars indicate beginning of electrical field stimulation and number of pulses applied). Scale bars are 10 µm.

**Figure S5 related to Figure 5 – Sandwich astrocyte plate preparation.** To plate rat astrocytes for sandwich co-culture the wells were pricked with a hot syringe needle to make little bumps on 4 corners. The plates were sterilized, coated (see suppl. methods) and astrocytes were plated on them.

**Supplemental tables**

**Table A: Primary antibodies (Cell culture and tissue)**

| **Antibody** | **Company** | **Host animal** | **Concentration** |
| --- | --- | --- | --- |
| Map2 | Abcam | Chicken | ICC & IHC – 1:500 |
| Synaptophysin1 | SYSY | Guinea pig | ICC – 1:1000; IHC – 1:500 |
| VGLUT1 | SYSY | Rabbit | ICC – 1:250 or 1:500; IHC – 1:250 |
| VGAT | SYSY | Rabbit | ICC – 1:500; IHC – 1:250 |
| Smi 312 | Covance | Mouse | ICC – 1:1000 |
| Homer1 | SYSY | Mouse | ICC – 1:100 |
| GAD65/67 | Millipore | Rabbit | ICC & IHC – 1:500 |
| PLZF | Calbiochem | Mouse | ICC – 1:50 |
| Pax6 | Millipore | Rabbit | ICC & IHC – 1:500 |
| Sox2 | Millipore | Rabbit | ICC – 1:1000 |
| Human Nucleus | Millipore | Mouse | IHC – 1:500 |
| Neurofilament | Sigma | Rabbit | IHC – 1:500 |
| OCT3/4 | Santa Cruz | Mouse | ICC – 1:1000 |
| Tra-1-60 | Santa Cruz | Mouse | ICC – 1:200 |
| Tra-1-81 | Millipore | Mouse | ICC – 1:250 |
| SSEA4 | Hybridomabank | Mouse | ICC – 1:50 |
| CTIP2 | Abcam | Rat | ICC – 1:250 |
| SATB2 | Abcam | Mouse | ICC – 1:25 |
| PROX1 | Millipore | Rabbit | ICC – 1:200 |
| Calbindin | Swant | Mouse | ICC – 1:100 |
| MEIS2 | Santa Cruz | Mouse | ICC – 1:50 |

**Table B: RT and Q-PCR primers**

| **Gene** | **PCR type** | **Forward primer** | **Reverse primer** | **Reference** |
| --- | --- | --- | --- | --- |
| Sox2 | RT-PCR | CATCACCCACAGCAAATGAC | TTTTTCGTCGCTTGGAGACT |  |
| Nestin | RT-PCR | CAGGAGAAACAGGGCCTACA | TAAGAAAGGCTGGCACAGGT |  |
| Pax6 | RT-PCR | CCGGCAGAAGATTGTAGAGC | CTCACACATCCGTTGGACAC |  |
| PLZF | RT-PCR | CTATGGGCGAGAGGAGAGTG | TCAATACAGCGTCAGCCTTG |  |
| Dach1 | RT-PCR | GTGGAAAACACCCCTCAGAA | CTTGTTCCACATTGCACACC |  |
| ZNF312 | RT-PCR | GCCTTCCACCAGGTCTACAA | GGTACAGGGAGGGAAGGAAG |  |
| HES5 | RT-PCR | GCCCGGGGTTCTATGATATT | GAGTTCGGCCTTCACAAAAG |  |
| CoupTF2 | Q-PCR | GGATGTGCTTCTAGGTGGTGA | AGGGAAAGAGTCAACTCGCC |  |
| PROX1 | Q-PCR | GTCCAATTGCCTTGTGTGCC | TCAAACGGCACTGAGCTTGT |  |
| SATB1 | Q-PCR | GTGCCTTGGGTAGTCCGC | AGAATCGGCGAGTGCAAAGTA |  |
| LHX6 | Q-PCR | TCGTTGAGGAGAAGGTGCTT | CGTCATGTCCGCTAGCTTCT | [1] |
| MEIS2 | Q-PCR | ACCAGTCAAATCGAGCAGTGA | GCTGACCATCCAACACAAAGC |  |
| GSX2 | Q-PCR | TGTCTCGACTCCGGAGGATT | TTCACTCGGCGGTTCTGAAA |  |
| ASCL1 | Q-PCR | CACCAACTGGTTCTGAGGGG | CGCCACTGACAAGAAAGCAC |  |
| VGAT | Q-PCR | GGACTCGTACGTGGCCATAG | AGCTCGATGATCTGCGCTAC |  |
| VGLUT1 | Q-PCR | TTCTGGCTGCTCGTCTCCTA | GGTTCATGAGTTTCGCGCTC |  |
| BF1 | Q-PCR | TGCCAAGTTTTACGACGGGA | AGGGTTGGAAGAAGACCCCT |  |
| TBR2 | Q-PCR | ACAACTATGATTCATCCCATCAGA | AGCGGGCTTGAGGTAAAGTG |  |
| PAX6 | Q-PCR | AGGTCAGGCTTCGCTAATGG | TGCTGATTGGTGATGGCTCA |  |
| CUX1 | Q-PCR | TTCTCACCAGCGGGGTTAAG | GCCGCTGGAGAAGAGAGTTT |  |
| BRN2 | Q-PCR | GTGCAAGCTGAAGCCTTTGT | CGCTGCGATCTTGTCTATGC |  |
| SATB2 | Q-PCR | GTGCCTTGGGTAGTCCGC | AGAATCGGCGAGTGCAAAGTA |  |
| TBR1 | Q-PCR | CAACAGCCTCCTGTCCAACT | GAACGGAGCTCCTTGGTAGG |  |
| CTIP2 | Q-PCR | CAGAGCAGCAAGCTCACG | GGTGCTGTAGACGCTGAA GG | [2] |
| HES5 | Q-PCR | AAGCACAGCAAAGCCTTCGT | CTGCAGGCACCACGAGTAG |  |
| DACH1 | Q-PCR | CAATGACTGCACCAACGCAA | TGCGGCATGATGTGAGAGTT |  |
| PLZF | Q-PCR | AGACGTACCTCTACCTGTGCT | TGTCATAGTCCTTCCTTCATCTCAC |  |
| SOX2 | Q-PCR | ATGGGTTCGGTGGTCAAGTC | CTGATCATGTCCCGGAGGTC |  |
| Eif4g2 | Q-PCR | AGGACCGCATGTTGGAGATT | TGAGGGGATGGATCCAACTTT |  |
| TDGF1 | RT-PCR | TGCTGCTCACAGGGCCCGATACTTC | TCCTTTCGAGCTCAGTGCACCACAAAAC | [3] |
| UTF1 | RT-PCR | CAGATCCTAAACAGCTCGCAGAAT | GCGTACGCAAATTAAAGTCCAGA | [3] |
| DNMT3B | RT-PCR | CAGGAGACCTACCCTCCACA | TGTCTGAATTCCCGTTCTCC | [4] |
| REX1 | RT-PCR | GCTGACCACCAGCACACTAGGC | TTTCTGGTGTCTTGTCTTTGCCCG | [4] |
| Sall4 | RT-PCR | GCCGTGAAGACCAATGAGAT | CTCCTTCCACGCAAGTTCTC | [4] |
| DPPA4 | RT-PCR | GGAGCCGCCTGCCCTGGAAAATTC | TTTTTCCTGATATTCTATTCCCAT | [3] |
| DPPA2 | RT-PCR | CCGTCCCCGCAATCTCCTTCCATC | ATGATGCCAACATGGCTCCCGGTG | [3] |
| ESG1 | RT-PCR | ATATCCCGCCGTGGGTGAAAGTTC | ACTCAGCCATGGACTGGAGCATCC | [3] |
| CD31 | RT-PCR | AACAGTGTTGACATGAAGAGCC | TGTAAAACAGCACGTCATCCTT | [5] |
| MAP2 | RT-PCR | CAGGTGGCGGACGTGTGAAAATTGAGAGTG | CACGCTGGATCTGCCTGGGGACTGTG | [3] |
| SOX17 | RT-PCR | GTGTGAATCTCCCCGACAG | TGTAACACTGCTTCTGGCC |  |
| Eif4g2 | RT-PCR | ATTCTTCGTTGTCAAGCCGCCAAAGTGGAG | AGTTGTTTGCTGCGGAGTTGTCATCTCGTC |  |

**Table C: Drugs in calcium imaging**

| **Name** | **Company** | **Working dilution** | **From stock** |
| --- | --- | --- | --- |
| Tetrodotoxin | Abcam | 2µM | 1mM in saline buffer |
| DNQX | Tocris | 10µM | 10mM in DMSO |
| Bicuculline | Sigma | 40µM | 10mM in water |
| AP5 | Ascent | 50µM | 5mM in water |

**Supplemental References**

1. Zhang Z, Gutierrez D, Li X, Bidlack F, Cao H, Wang J, et al. The LIM homeodomain transcription factor LHX6: a transcriptional repressor that interacts with pituitary homeobox 2 (PITX2) to regulate odontogenesis. J Biol Chem. 2013;288(4):2485-500. doi: 10.1074/jbc.M112.402933. PubMed PMID: 23229549; PubMed Central PMCID: PMCPMC3554917.

2. Ip BK, Bayatti N, Howard NJ, Lindsay S, Clowry GJ. The corticofugal neuron-associated genes ROBO1, SRGAP1, and CTIP2 exhibit an anterior to posterior gradient of expression in early fetal human neocortex development. Cereb Cortex. 2011;21(6):1395-407. doi: 10.1093/cercor/bhq219. PubMed PMID: 21060114; PubMed Central PMCID: PMCPMC3097990.

3. Takahashi K, Tanabe K, Ohnuki M, Narita M, Ichisaka T, Tomoda K, et al. Induction of pluripotent stem cells from adult human fibroblasts by defined factors. Cell. 2007;131(5):861-72. doi: 10.1016/j.cell.2007.11.019. PubMed PMID: 18035408.

4. Plews JR, Li J, Jones M, Moore HD, Mason C, Andrews PW, et al. Activation of pluripotency genes in human fibroblast cells by a novel mRNA based approach. PLoS One. 2010;5(12):e14397. doi: 10.1371/journal.pone.0014397. PubMed PMID: 21209933; PubMed Central PMCID: PMCPMC3012685.

5. Kurian L, Sancho-Martinez I, Nivet E, Aguirre A, Moon K, Pendaries C, et al. Conversion of human fibroblasts to angioblast-like progenitor cells. Nat Methods. 2013;10(1):77-83. Epub 2012/12/04. doi: 10.1038/nmeth.2255. PubMed PMID: 23202434; PubMed Central PMCID: PMCPmc3531579.
